# A genome-wide association study of survival in patients with sepsis

**DOI:** 10.1186/s13054-022-04208-5

**Published:** 2022-11-05

**Authors:** Tamara Hernandez-Beeftink, Beatriz Guillen-Guio, Jose M. Lorenzo-Salazar, Almudena Corrales, Eva Suarez-Pajes, Rui Feng, Luis A. Rubio-Rodríguez, Megan L. Paynton, Raquel Cruz, M. Isabel García-Laorden, Miryam Prieto-González, Aurelio Rodríguez-Pérez, Demetrio Carriedo, Jesús Blanco, Alfonso Ambrós, Elena González-Higueras, Elena Espinosa, Arturo Muriel, Eduardo Tamayo, María M. Martin, Leonardo Lorente, David Domínguez, Abelardo García de Lorenzo, Heather M. Giannini, John P. Reilly, Tiffanie K. Jones, José M. Añón, Marina Soro, Ángel Carracedo, Louise V. Wain, Nuala J. Meyer, Jesús Villar, Carlos Flores, the Genetics of Sepsis (GEN-SEP) Network

**Affiliations:** 1 Research Unit, Hospital Universitario Nuestra Señora de Candelaria, Carretera del Rosario S/N, Santa Cruz de Tenerife, Spain; 2 Research Unit, Hospital Universitario de Gran Canaria Dr. Negrin, Las Palmas de Gran Canaria, Spain; 3 Department of Health Sciences, University of Leicester, Leicester, UK; 4 Genomics Division, Instituto Tecnológico y de Energías Renovables (ITER), Santa Cruz de Tenerife, Spain; 5 CIBER de Enfermedades Respiratorias, Instituto de Salud Carlos III, Madrid, Spain; 6 Department of Biostatistics, Epidemiology, and Informatics, University of Pennsylvania Perelman School of Medicine, Philadelphia, USA; 7 Genomic Medicine Group, Biomedical Research Center of Rare Diseases (CIBERER), University of Santiago de Compostela, Santiago de Compostela, Spain; 8 Intensive Care Unit, Complejo Asistencial Universitario de Palencia, Palencia, Spain; 9 Department of Anesthesiology, Hospital Universitario de Gran Canaria Dr. Negrín, Las Palmas de Gran Canaria, Spain; 10 Department of Medical and Surgical Sciences, University of Las Palmas de Gran Canaria, Gran Canaria, Spain; 11 Intensive Care Unit, Complejo Hospitalario Universitario de León, León, Spain; 12 Intensive Care Unit, Hospital Universitario Rio Hortega, Valladolid, Spain; 13 Intensive Care Unit, Hospital General de Ciudad Real, Ciudad Real, Spain; 14 Intensive Care Unit, Hospital Virgen de la Luz, Cuenca, Spain; 15 Department of Anesthesiology, Hospital Universitario N.S. de Candelaria, Santa Cruz de Tenerife, Spain; 16 CIBER de Enfermedades Infecciosas, Department of Anesthesiology and Resuscitation, Hospital Clínico Universitario de Valladolid, Valladolid, Spain; 17 Departamento de Cirugía, Facultad de Medicina, Universidad de Valladolid, Valladolid, Spain; 18 Intensive Care Unit, Hospital Universitario Nuestra Señora de Candelaria, Santa Cruz de Tenerife, Spain; 19 Intensive Care Unit, Hospital Universitario de Canarias, La Laguna, Tenerife Spain; 20 Intensive Care Unit, Hospital Universitario La Paz, IdiPAZ, Madrid, Spain; 21 Division of Pulmonary, Allergy, and Critical Care Medicine, University of Pennsylvania Perelman School of Medicine, Philadelphia, USA; 22 Department of Anesthesiology, Hospital Clinico Universitario de Valencia, Valencia, Spain; 23 Genomic Medicine Group, CIMUS, University of Santiago de Compostela, Santiago de Compostela, Spain; 24 Galician Foundation of Genomic Medicine, Foundation of Health Research Institute of Santiago de Compostela (FIDIS), SERGAS, Santiago de Compostela, Spain; 25 Leicester Respiratory Biomedical Research, Centre, National Institute for Health Research, Glenfield Hospital, Leicester, UK; 26 Li Ka Shing Knowledge Institute, St. Michael’s Hospital, Toronto, ON Canada; 27 Facultad de Ciencias de la Salud, Universidad Fernando Pessoa Canarias, Las Palmas de Gran Canaria, Spain

**Keywords:** Sepsis, Genome-wide association study, Genomics, 28 days, Outcome

## Abstract

**Background:**

Sepsis is a severe systemic inflammatory response to infections that is accompanied by organ dysfunction and has a high mortality rate in adult intensive care units. Most genetic studies have identified gene variants associated with development and outcomes of sepsis focusing on biological candidates. We conducted the first genome-wide association study (GWAS) of 28-day survival in adult patients with sepsis.

**Methods:**

This study was conducted in two stages. The first stage was performed on 687 European sepsis patients from the GEN-SEP network and 7.5 million imputed variants. Association testing was conducted with Cox regression models, adjusting by sex, age, and the main principal components of genetic variation. A second stage focusing on the prioritized genetic variants was performed on 2,063 ICU sepsis patients (1362 European Americans and 701 African-Americans) from the MESSI study. A meta-analysis of results from the two stages was conducted and significance was established at *p* < 5.0 × 10^−8^. Whole-blood transcriptomic, functional annotations, and sensitivity analyses were evaluated on the identified genes and variants.

**Findings:**

We identified three independent low-frequency variants associated with reduced 28-day sepsis survival, including a missense variant in *SAMD9* (hazard ratio [95% confidence interval] = 1.64 [1.37–6.78], *p* = 4.92 × 10^−8^). *SAMD9* encodes a possible mediator of the inflammatory response to tissue injury.

**Interpretation:**

We performed the first GWAS of 28-day sepsis survival and identified novel variants associated with reduced survival. Larger sample size studies are needed to better assess the genetic effects in sepsis survival and to validate the findings.

**Supplementary Information:**

The online version contains supplementary material available at 10.1186/s13054-022-04208-5.

## Introduction

Sepsis is defined as a severe systemic inflammatory response to infections that is accompanied by organ dysfunction [1]. It is recognized as a global priority and its incidence in adults is estimated at approximately 189 cases per 100,000 people per year [2, 3]. In the intensive care units (ICUs), sepsis is associated with an overall mortality rate of about 30% [4, 5] and with significant morbidity in survivors. The risk of death in patients with sepsis increases with hemodynamic instability (i.e., septic shock) or due to respiratory complications, such as the acute respiratory distress syndrome (ARDS) [6]. Given the lack of specific therapeutic options and the underlying etiological complexity, multiple studies have focused on improving prevention, diagnosis, and prognosis of sepsis [5].

Genetic variation influences the host immune response to microbial agents [7–10]. In this sense, the genome-wide association studies (GWAS) have an enormous potential to reveal genetic factors for disease susceptibility, severity, and/or survival, as it has been shown for several infectious diseases [10–14]. However, the number of GWAS of sepsis or its complications is limited. To date, only four GWAS have been completed for sepsis mortality, although the likelihood of death for each patient over time was not considered [15–18]. Specifically, Rautanen and colleagues analyzed 28-day mortality in patients with pneumonia, linking the FER tyrosine kinase (*FER*) gene variation with reduced risk for death from sepsis [15]. Nevertheless, another study was unable to replicate this finding in independent patients [19]. Additionally, Scherag and colleagues found that the top ranking variants associated with 28-day mortality from sepsis in their study were located in the Vacuolar Protein Sorting 13 Homolog A (*VPS13A*) gene [16]. Rosier and colleagues identified variants within the Cytokine Inducible SH2 Containing Protein (*CISH*) gene associated with mortality due to septic shock at day 7 or day 28 [17]. Finally, D’Urso and colleagues performed a GWAS of susceptibility and mortality in septic shock and polygenic risk score (PRS) analysis to assess the genetic overlap between septic shock risk/mortality with clinically relevant traits. In the 28-day mortality GWAS analysis, they found association of an intronic variant in the Collagen Type IV Alpha 2 Chain (*COL4A2*) gene [18].

Based on all the above-mentioned evidence, here we performed a GWAS of 28-day sepsis survival to identify novel genetic variants associated with sepsis outcome taking into account the probability estimates of death for each patient over time.

## Methods

### Study design and participants

**Fig. 1:**
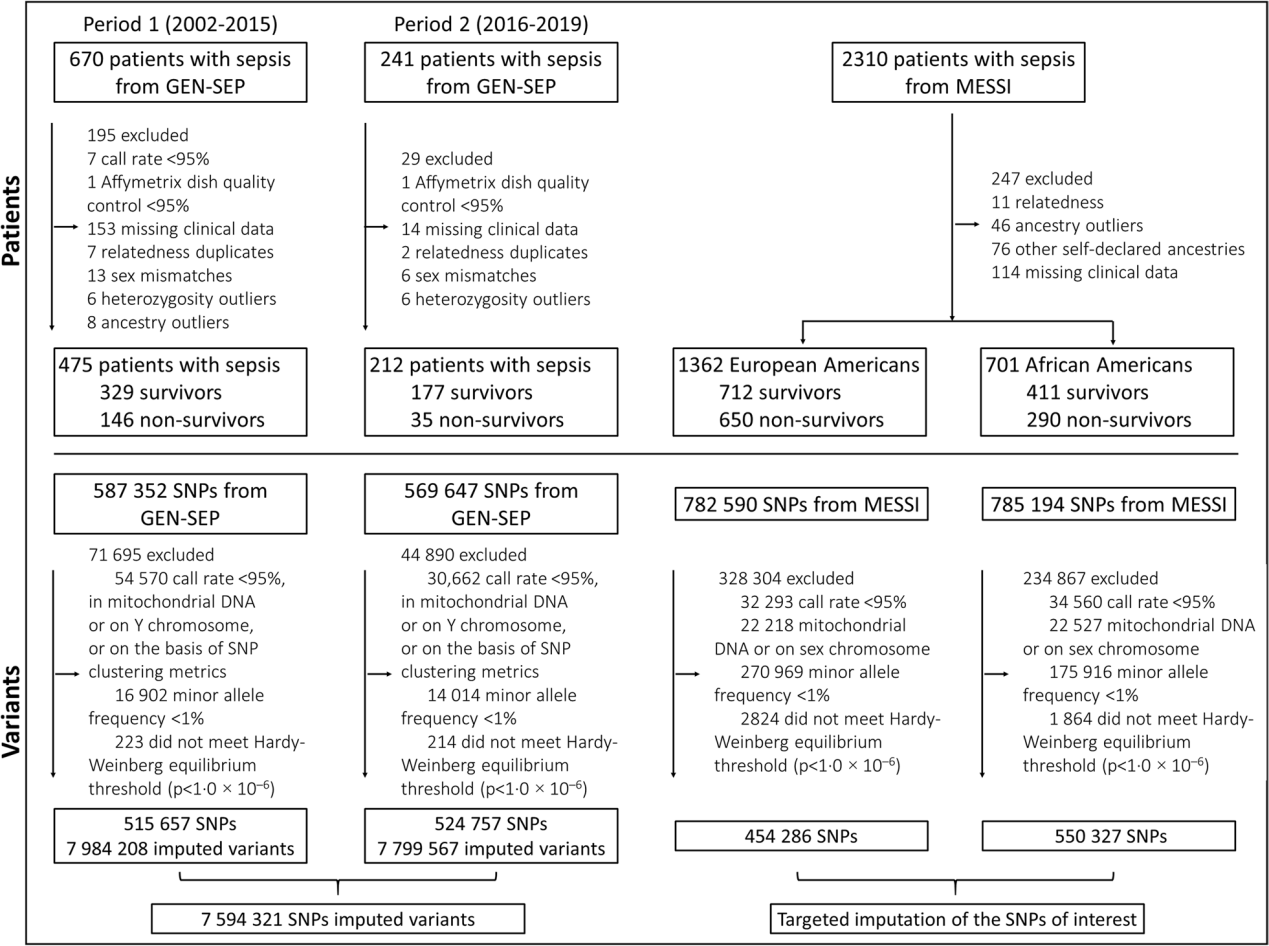
Study profile. SNPs, single-nucleotide polymorphisms

We performed a GWAS of 28-day survival in patients with sepsis. This study was conducted in two stages. The first stage was based on two cohorts of patients from the GEN-SEP study [20], where association results were meta-analyzed and used to prioritize variants (Fig. 1). In the second stage, we followed up these variants in independent sepsis patients of European and African-American ancestry from the MESSI study [21]. Finally, a meta-analysis of results of the 2750 patients (1121 non-survivors) from the two stages was done and genome-wide significance was established at *p* < 5.0 × 10^−8^.

The GEN-SEP study, used for the first stage, included 475 (146 non-survivors) European ancestry patients considered for a previous GWAS of sepsis-associated ARDS [20] recruited between 2002 and 2015 for which follow-up records of 28-day survival were available (1st GEN-SEP period). In a second round of patient recruitment, between 2016 and 2019, another 212 (35 non-survivors) patients were included in the GEN-SEP study (2nd GEN-SEP period) (Fig. 1) (see supplementary material for further details). Sepsis diagnosis was clinically defined according to the Third International Consensus Definitions for Sepsis [1].

The MESSI study [21], used for the second stage, included a total of 1362 (650 non-survivors) unrelated European Americans and 701 (290 non-survivors) African-Americans with a period of recruitment from 2008 to 2019 (see supplementary material for further details).

All participating studies were done according to The Code of Ethics of the World Medical Association (Declaration of Helsinki), and written informed consent was obtained from all participants or their representatives. The Research Ethics Committees at all participating centers approved this study.

### Genotyping and statistical analyses

Genotyping in the GEN-SEP was performed using the Axiom Genome-Wide Human CEU 1 array (Affymetrix, Santa Clara, CA, USA). Genotyping quality control procedures are detailed in supplementary material (Fig. 1). We also calculated the main axis of genetic variation using principal component (PC) analyses (Additional file 1: Figure S1). In MESSI, SNPs were genotyped using the Affymetrix Axiom TxArray v.1 (Affymetrix) (see supplementary material for further details).

In the first stage, we used Cox proportional hazards model to take into account time-to-event in the analysis of the genetic associations adjusting for age, sex, and the first two main PCs. Results were obtained for a total of 7,872,728 (1st GEN-SEP period) and 7,829,916 (2nd GEN-SEP period) SNPs. An intersection of 7,682,187 SNPs was considered for fixed-effect model meta-analysis. Variants were prioritized for the next stage if they satisfied having the same effect direction, a *p* < 0.05 in both GEN-SEP recruitment periods, and a *p* < 5.0 × 10^−7^ after the meta-analysis of both periods. Association results of this first stage were also inspected to evaluate whether the variants or genes previously associated with sepsis mortality by other studies were also associated in GEN-SEP [15–18].

In the second stage, the prioritized independent variants were tested for association in the MESSI European Americans and African-Americans, separately, using Cox regression models, also considering sex, age, and the first two main PCs as covariates.

Finally, a fixed-effect model meta-analysis from the GEN-SEP and MESSI studies was performed, and the genome-wide significance was declared at *p* < 5.0 × 10^−8^. A sensitivity analysis was conducted for the genome-wide significant variants, adjusting the models for different clinical and demographic variables and the index event bias. We also used the Nagelkerke’s *R*^2^ to estimate the proportion of variance explained by the independent most significant (sentinel) variants by separate or when combined into a PRS. More details are included in supplementary material.

### Association studies in the HLA genes

Given the importance of the major histocompatibility complex (MHC) in inflammatory and immunological diseases, we performed association testing with 28-day sepsis survival of genetic variation in the HLA region. Association analyses were performed only on the GEN-SEP cohort by using Cox regressions adjusting for sex, age, and the main two PCs. This analysis was restricted to 207 classic HLA alleles and 1034 amino acids that had a frequency > 1%. Considering the multiple tests adjustment, significance thresholds for the HLA analysis were set at *p* < 2.49 × 10^−4^ and *p* < 4.83 × 10^−5^, respectively.

### Annotation of the functional effects of associated variants and gene expression

The functional effects of the sentinel variants, their linkage disequilibrium proxies (*r*^2^ > 0.7), and related genes were assessed based on empirical data from different integrated software tools and datasets (see supplementary material for further details). To assess differential gene expression of the genes near the sentinel variants, we accessed the public gene expression datasets GSE54514, GSE65682, and GSE32707, containing data for sepsis survival and sepsis-associated ARDS.

### Polygenic risks of sepsis and effects on 28-day sepsis survival

We examined whether the polygenic component of sepsis risk was associated with 28-day sepsis survival through PRS (see supplementary material for further details).

First, we obtained a model of the genetic risk for sepsis by a GWAS of all available sepsis cases from GEN-SEP and population controls (Additional file 1: Table S1) using logistic regressions adjusted by sex, age, and the two main PCs.

Then, we constructed the PRS for sepsis risk by including in the score those variants that met a *p* value threshold in the sepsis risk GWAS and varied this threshold to investigate the effect of including more variants in the score.

Finally, we tested if the score was associated with 28-day survival among GEN-SEP patients, adjusting for sex, age, and two main PCs. For this, we used Cox regression and established the *p* < 0.001 threshold for defining significant associations of the risk score.

Additionally, we performed sensitivity analyses to assess the sepsis risk score association with 28-day sepsis survival after (1) excluding variants that deemed significantly associated with sepsis in candidate gene studies (Additional file 1: Table S2), and (2) excluding variants significantly associated with sepsis mortality in previous GWAS (see supplementary material for further details).

## Results

**Fig. 2:**
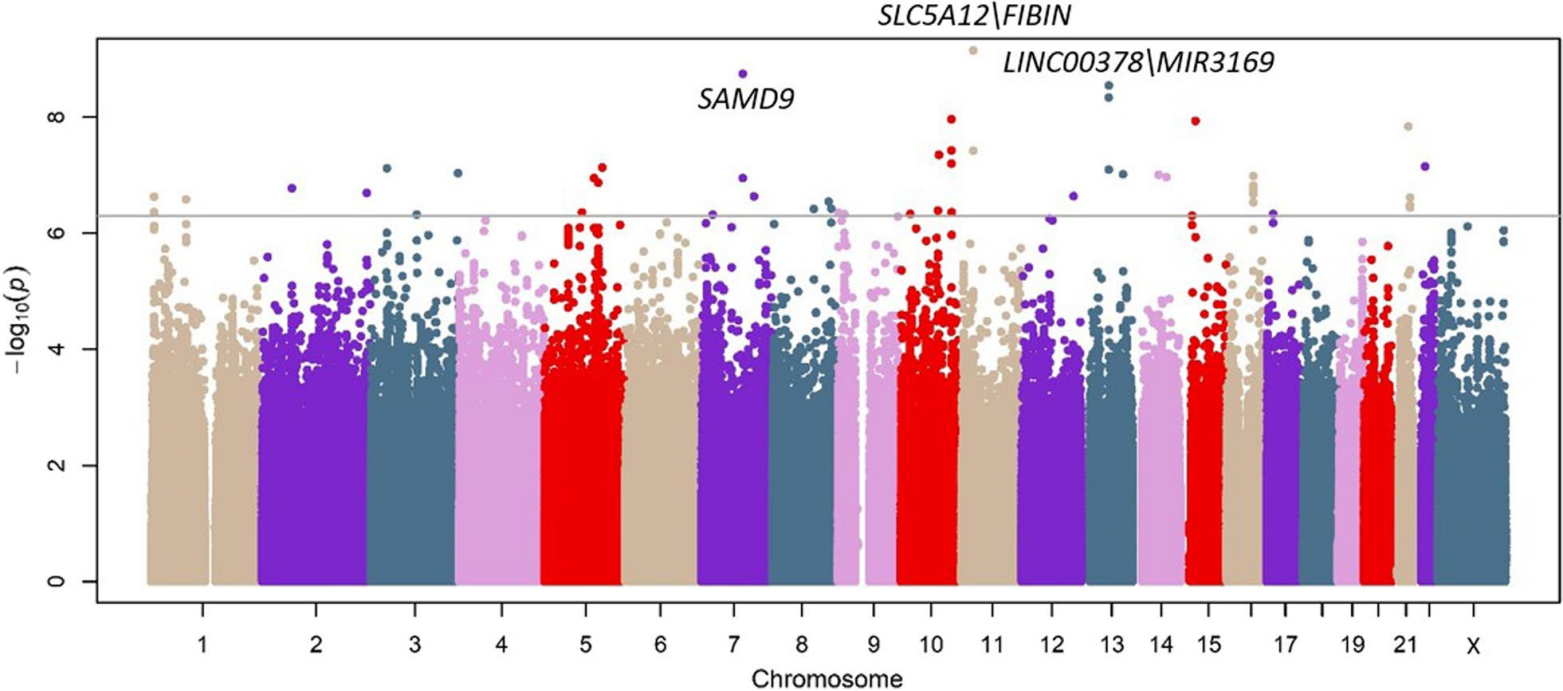
Association results for the 28-day sepsis survival for the first stage. Manhattan plot representing in the *x*-axis the genomic positions and in the *y*-axis the significance (− log10(*p* value)). The horizontal line indicates the significance threshold for prioritization to the second stage (*p* = 5.0 × 10^−7^)

Demographic and clinical features of patients from the first stage are described in Additional file 1: Table S3. After association testing, 11 independent variants were prioritized in the first stage (Fig. 2; Additional file 1: Table S4). The genomic inflation factor of the results from this stage (*λ* = 1.06) did not indicate major systematic deviations from the null hypothesis of no association (Additional file 1: Figure S2).

In the second stage, we were able to follow up on the association of 10 of the 11 prioritized variants (Additional file 1: Table S4). Three of these variants reached the genome-wide significance threshold in the meta-analysis (Table 1): a missense variant in the Sterile Alpha Motif Domain containing 9 (*SAMD9*) (rs34896991; p.Ala1556Thr), an intergenic variant to Solute Carrier family 5 member 12 (*SLC5A12*) and Fin Bud Initiation Factor Homolog (*FIBIN*) genes (rs146257041), and an intergenic variant between two non-coding RNAs (LINC00378 and MIR3169) (rs138347802). These three SNPs were of low frequency, with a MAF of ~ 1% in the study population. Regional association results and Kaplan–Meier survival plots for the three genome-wide significant variants in the GEN-SEP are reported in supplementary material (Additional file 1: Figures S3 and S4). Results remained robust in the sensitivity analyses (Table 2; Additional file 1: Table S5). Based on Nagelkerke’s *R*^2^, the models for the sentinel variants explain separately nearly 6% of variance, whereas a PRS combination of these three variants explains nearly 9% (Additional file 1: Table S6). These results must be taken with caution because of the potential overfitting of the models.

**Table 1:** Prioritized independent SNPs from the first stage of the GWAS of 28-day sepsis survival

rsID	Position (Hg19)	A1/A2	Func.Gene	Gene	GEN-SEP (*N* = 687)			MESSI (*N* = 1362) (European Americans)		MESSI (*N* = 701) (African-Americans)		Meta-analysis (*N* = 2750)	
					EAF	HR (95% CI)	*p* value	HR (95% CI)	*p* value	HR (95% CI)	*p* value	HR (95% CI)	*p* value
rs34896991	7:92,730,745	C/T	Exonic	*SAMD9*	0.018	4.75 (2.86–7.89)	1.77 × 10^−9^	0.61 (0.29–1.28)	0.188	1.44 (0.46–4.50)	0.534	1.64 (1.37–6.78)	4.92 × 10^−8^
rs146257041	11:26,983,813	A/G	Intergenic	*SLC5A12\FIBIN*	0.015	5.14 (3.06–8.65)	7.04 × 10^−10^	NA	NA	0.89 (0.12–6.39)	0.911	4.59 (2.77–7.59)	3.00 × 10^−9^*
rs138347802	13:61,367,197	A/G	Intergenic	LINC00378\MIR3169	0.014	8.33 (4.1–16.91)	4.56 × 10^−9^	0.68 (0.30–1.52)	0.345	2.98 (0.95–9.38)	0.062	2.57 (1.82–13.03)	4.44 × 10^−8^

A1, Ref allele; A2, Effect allele, *EAF* effect allele frequency, *HR* hazard ratio, *CI* confidence interval, Position: Genomic coordinates

*Meta-analysis performed between two studies

**Table 2:** Sensitivity analyses in the GEN-SEP study of the three genome-wide significant variants. The models adjusted for the indicated variables plus gender, age, and the two main principal components

	*N*	rs34896991		rs146257041		rs138347802	
		HR (95% CI)	*p* value	HR (95% CI)	*p* value	HR (95% CI)	*p* value
BMI	364	6.68 (3.22–13.86)	3.47 × 10^−7^	5.64 (2.10–15.18)	6.12 × 10^−4^	5.72 (0.72–45.32)	0.098
SAPS	264	4.06 (1.54–10.72)	4.66 × 10^−3^	9.95 (4.55–21.74)	8.32 × 10^−9^	8.63 (1.86–40.01)	5.90 × 10^−3^
APACHE II	672	3.48 (2.05–5.91)	3.66 × 10^−6^	5.84 (3.43–9.94)	7.39 × 10^−11^	8.65 (4.27–17.53)	2.07 × 10^−9^
Comorbidities$	600	4.24 (2.46–7.33)	2.19 × 10^−7^	5.39 (3.09–9.40)	2.91 × 10^−9^	7.60 (3.14–18.36)	6.69 × 10^−6^
SOFA	661	4.58 (2.72–7.69)	9.49 × 10^−9^	5.10 (2.99–8.71)	2.40 × 10^−9^	8.50 (4.16–17.39)	4.59 × 10^−9^
Sepsis of pulmonary origin	681	4.32 (2.62–7.13)	9.74 × 10^−9^	4.71 (2.78–8.00)	9.33 × 10^−9^	8.08 (3.98–16.43)	7.54 × 10^−9^
*Pathogen, %*							
Gram-positive	487	4.97 (2.68–9.20)	3.51 × 10^−7^	5.36 (3.02–9.51)	9.82 × 10^−9^	13.11 (5.01–34.32)	1.61 × 10^−7^
Gram-negative	487	4.97 (2.68–9.21)	3.42 × 10^−7^	5.32 (3.00–90.46)	1.18 × 10^−8^	13.17 (5.04–34.43)	1.47 × 10^−7^
Others*	487	4.91 (2.64–9.13)	4.73 × 10^−7^	5.13 (2.89–9.12)	2.30 × 10^−8^	12.91 (4.93–33.85)	1.96 × 10^−7^

*APACHE II* acute physiology and chronic health evaluation II, *BMI* Body mass index, *ICU* intensive care unit, *SAPS* simplified acute physiology score II, *SOFA* sequential organ failure assessment

*Includes: mixed Gram-positive and Gram-negative infection, fungi, virus, and polymicrobial

^$^Includes: cancer, age > 80 years old, hepatopathy, valvular disease, immunodeficiency, severe brain damage, morbid obesity, chronic disease, autoimmune disease, pregnancy, myopathy, pneumonia, and serious recurrent infections

Finally, we tested previously associated variants with sepsis mortality in the results of the first stage. We found that leading variants of previous GWAS [15–18] were not replicated in the GEN-SEP study (*FER* rs4957796, Hazard Ratio [HR]: 1.09 [95% CI = 0.83–1.43], *p* = 0.550; *VPS13A* rs117983287, HR: 0.36 [95% CI = 0.09–1.48], *p* = 0.158; *CISH* rs143356980, HR: 1.42 [95% CI = 0.61–3.33], *p* = 0.419; and *COL4A2* rs368584, HR: 1.02 [95% CI = 0.83–1.26], *p* = 0.817) (Additional file 1: Table S7). Similarly, an assessment of the association results in and around (± 50 kb) the corresponding genes (*FER, VPS13A, CISH,* and *COL4A2*) did not reveal any significant finding (Supplementary Methods; Table S7). Likewise, none of classical HLA alleles (*p*_lowest_ = 0.0169) or amino acids (*p*_lowest_ = 0.0169) was significantly associated with sepsis survival after multiple testing adjustments (Additional file 1: Figure S5; Table S8), suggesting that common genetic variation at the HLA is not a major driver of sepsis survival or has a modest effect size that could not be detected with the current design.

### Potential biological effects of the 28-day sepsis survival-associated variants

We then explored the potential functional implications of the three genome-wide significant variants, rs34896991 in *SAMD9*, rs146257041 intergenic to *SLC5A12*\*FIBIN*, and rs138347802 intergenic to LINC00378\MIR3169. Based on distinct functional annotations, we observed a few regulatory activities linked to all three overlapped promotor or enhancer regions in multiple cell types including blood cells, and T cells (Additional file 1: Table S9). According to GTEx data, LINC00378 and MIR3169 are expressed only in testis, *SLC5A12* is mainly expressed in the kidney and in the small intestine, *FIBIN* is mainly expressed in arteries (aorta and tibial), tibial nerve, and vagina, while *SAMD9* is expressed broadly across many tissues, but mainly in the esophagus (mucosa), in transformed lymphocytes, and in whole blood. Based on the IPF Cell Atlas in control donors (information was absent for MIR3169), *SAMD9* is expressed ubiquitously across the different cell types, whereas the *SLC5A12, FIBIN*, and LINC00378 expression is restricted to a few cell types including vascular, muscular, epithelial, and immune (Additional file 1: Figure S6).

No significant expression quantitative trait loci (eQTLs) were identified in GTEx for rs34896991 and rs138347802. Nevertheless, four significant eQTLs were found in brain and testis for rs146257041. None of these three variants obtained a significant score predicted using DeepSEA. A scan for previously reported trait associations for the three variants based on PhenoScanner found that rs34896991 in *SAMD9* was also associated with the cause of death in other specified degenerative diseases of the nervous system (Additional file 1: Table S9). Interestingly, other variants in *SAMD9*, *SLC5A12*, *FIBIN*, and in the non-coding RNA (LINC00378) have also been associated with different causes of death according to PhenoScanner results. Regarding the non-coding RNAs, LINC00378 has Cyclin-Dependent Kinase Inhibitor 1A (*CDKN1A*) as the main target and is linked to different types of cancers, while MIR3169 targets genes that are mainly involved in the p53 signaling pathway.

To further evaluate the biological implications of the genes near the identified GWAS loci, whole-blood transcriptomic array data from sepsis survivors and non-survivors were assessed. While information was only available for coding genes, an upregulation of *SAMD9* expression in non-surviving sepsis patients was observed in GSE54514 (log fold change: 0.545 adjusted FDR *p* value: 2.18 × 10^−3^) (Additional file 1: Figure S7). Nevertheless, this gene expression difference among the sepsis patient groups was unrelated to the presence of ARDS (log fold change: 0.011; adjusted FDR *p* value: 0.996) (Additional file 1: Figure S8). A final assessment of transcriptomic array data from independent sepsis survivors and non-survivors from GSE65682 did not validate the association of *SAMD9* expression with sepsis survival (log fold change: − 0.01 *p* value: 0.688) (Additional file 1: Figure S7).

### Polygenic risks of sepsis and effects on 28-day sepsis survival

Finally, we used PRS to investigate whether the polygenic component of sepsis risk was associated with 28-day sepsis survival. We found that the sepsis risk PRS was not significantly associated with sepsis survival at any of the cutoffs (Additional file 1: Figure S9). These results were similar when the models excluded the variants significantly associated with sepsis in the previous candidate gene studies or in the sepsis mortality GWAS.

## Discussion

To our knowledge, we report the first GWAS of 28-day sepsis survival conducted to date. In addition, given the importance of the MHC in inflammatory and immunological diseases, we also assessed for the first time the association of the classical HLA alleles and amino acids with 28-day sepsis survival. Our findings revealed three novel low-frequency variants associated with reduced 28-day survival among sepsis patients: one in *SAMD9* (the p.Ala1556Thr exonic variant), one intergenic to *SLC5A12\FIBIN*, and another intergenic to LINC00378\MIR3169. The functional annotation analyses revealed a modest regulatory activity of the sentinel variants. Besides, we found inconsistent results in the association of *SAMD9* expression in whole blood in two independent cohort studies. We were unable to replicate the findings from previous GWAS of sepsis mortality as has been observed in other studies [18, 19]. We also found a lack of overlap between the polygenic component of sepsis risk and sepsis survival. Taken together, while these results are promising, they should be taken with caution given that they only explain a small proportion of the outcome (likely less than our estimates when tested in independent studies) and the studies will require hundreds of thousands of participants to optimally assess genetic effects in the entire allele frequency spectrum and to tackle the underlying complexities of sepsis.

*SAMD9* could play a critical role in the inflammatory response during tissue injury and apoptosis [22, 23]. This gene encodes one of the SAM domain-containing proteins that has diverse roles for cellular processes via polymerization and participates in protein interactions and RNA binding [24–26]. It has been observed that the *SAMD9* upregulation triggered an accumulation of macrophages increasing low-grade glioma progression [27]. Linked to this, it was observed that *SAMD9* interacts with Ral Guanine Nucleotide Dissociation Stimulator Like 2 (RGL2) to decrease the expression of Early growth response protein 1 (EGR1) [28], which is a key regulator of inflammation in human macrophages [29]. *SAMD9* has been found to be significantly upregulated in vivo in peripheral blood mononuclear cells during inflammation and in vitro during T cell activation, and its expression is regulated at both the genetic and epigenetic levels [30]. Therefore, SAMD9 could serve as a T cell activation marker acting as an anti-inflammatory factor [30]. The osmotic shock and interferon-gamma (IFN-γ) tightly regulate *SAMD9* expression [28, 31, 32]. In fact, Chefetz and colleagues observed that SAMD9 was upregulated by tumor necrosis factor-alpha (TNF-α) through p38 mitogen-activated protein kinases (p38 MAPKs) and nuclear factor-kappa-B (NF-κB) [31]. Moreover, mutations in *SAMD9* have been linked to immunodeficiency, neutropenia, impaired anti-cytomegalovirus response, and gastrointestinal disorder [33], and to severe multisystem disorders and complex phenotypes characterized by recurrent infection, dysphagia, and profound deafness [34]. *SAMD9* is also strongly associated with mean corpuscular hemoglobin or volume and red cell distribution width [35, 36]. Therefore, we could hypothesize that the *SAMD9* upregulation, which is inducible by various inflammatory, immunological, and stress factors, might activate T cells and produce the accumulation of macrophages through its interaction with RGL2, thus conferring protection against the systemic dysregulation that occurs during sepsis. This would also be supported by its anti-tumoral, anti-inflammatory, and anti-viral activity. Because the T allele of rs34896991 predicts a missense change in *SAMD9*, this allele could act as a defective variant explaining its association with increased mortality risk among the patients with sepsis.

Our results also revealed two intergenic variants significantly associated with sepsis survival. One of them was located between *SLC5A12* and *FIBIN* genes. In particular, *SLC5A12* encodes an apical cell membrane protein that acts as a metabolite transporter involved in the nuclear factor E2-related factor 2 (NRF2) pathway [37, 38] which is key in response to oxidative stress [39–41]. FIBIN is a secreted protein acting downstream of retinoic acid and Wnt signaling [42, 43]. The other intergenic variant was located between a long non-protein coding RNA (LINC00378) and a microRNA (MIR3169). However, the functional information of these two non-coding genes is scarce, both linked to the cellular response to DNA damage.

We acknowledge some strengths and limitations of the study. Among the strengths, the results were supported by two independent geographically distinct studies with diverse ancestries. In addition, both cohorts were prospectively enrolled using consensus criteria for sepsis. Because of that, a robust sensitivity analysis was possible to control for potential confounders. Linked to this, although the significance and the effect direction of the associated variants were not affected by the index event bias correction, all the cases used in the survival analysis were also used to assess sepsis risk and this constitutes a limitation for the approach. The main weaknesses of the study are that we could not assess rarer or structural variants in the analyses. Other approaches such as exome or whole-genome sequencing are needed to analyze the role of these rare genetic variants. In addition, further functional characterization of the prioritized variants will be needed to further dissect the mechanistic connections with the pathophysiology of sepsis.

## Conclusion

In conclusion, we have completed a GWAS of 28-day sepsis survival and have identified three novel variants associated with reduced survival, one of them involving a missense variant. Given that the three variants only explain a small proportion of the outcome, more studies with thousands of participants will be needed to optimally assess the genetic effects in sepsis survival and to further validate our findings.

## Supplementary Information

**Additional file 1. Supplementary methods, figures, and tables.**The supplementary methods include a description of the GEN-SEP and MESSI study populations, genotyping and quality control, more details of the genome-wide association study of 28-day sepsis survival and the index event bias assessment, tools for the annotation of the functional effects of the sentinel variants and the gene expression of related genes, the association of polygenic risks of sepsis with the 28-day sepsis survival, the replication of genes from previous sepsis mortality GWAS, and related references.
The supplementary figures include a principal component analysis, the quantile-quantile plot of the GWAS, regional plots for the genome-wide significant variants, Kaplan-Meier 28-day survival plots, a manhattan plot of sepsis 28-day survival association study results in the HLA region, a plot of expression of related genes across cell types, a boxplot of SAMD9 gene expression values in GSE54514 and GSE65682, a boxplot of SAMD9 gene expression values in GSE32707, and polygenic risk score (PRS) model fitting.
The supplementary tables show the relevant demographic and clinical features of cases and controls used for the GWAS of sepsis risk and for the GEN-SEP patients, the list of previous candidate genes associated with sepsis risk, the prioritized independent SNPs, the index bias event results, the association results with 28-day mortality and the percentage of variation explained by the sentinel SNPs by separate or as part of a PRS, the association results of the sentinel variants from other sepsis mortality GWAS studies, the nominally significant results for the classical HLA alleles and amino acids, and the functional assessment of variants associated with 28-day sepsis survival.

## Data Availability

The data used and analyzed during this study are available from the corresponding author on reasonable request.
