# Supplementary material for: A genome-wide association study of survival in patients with sepsis
Source: Crit Care. 2022 Nov 5;26:341. doi: 10.1186/s13054-022-04208-5 (PMC9637317; doi:10.1186/s13054-022-04208-5)
Supplement: Supplementary file 1 — Additional file 1. Supplementary methods, figures, and tables.The supplementary methods include a description of the GEN-SEP and MESSI study populations, genotyping and quality control, more details of the genome-wide association study of 28-day sepsis survival and the index event bias assessment, tools for the annotation of the functional effects of the sentinel variants and the gene expression of related genes, the association of polygenic risks of sepsis with the 28-day sepsis survival, the replication of genes from previous sepsis mortality GWAS, and related references. The supplementary figures include a principal component analysis, the quantile-quantile plot of the GWAS, regional plots for the genome-wide significant variants, Kaplan-Meier 28-day survival plots, a manhattan plot of sepsis 28-day survival association study results in the HLA region, a plot of expression of related genes across cell types, a boxplot of SAMD9 gene expression values in GSE54514 and GSE65682, a boxplot of SAMD9 gene expression values in GSE32707, and polygenic risk score (PRS) model fitting. The supplementary tables show the relevant demographic and clinical features of cases and controls used for the GWAS of sepsis risk and for the GEN-SEP patients, the list of previous candidate genes associated with sepsis risk, the prioritized independent SNPs, the index bias event results, the association results with 28-day mortality and the percentage of variation explained by the sentinel SNPs by separate or as part of a PRS, the association results of the sentinel variants from other sepsis mortality GWAS studies, the nominally significant results for the classical HLA alleles and amino acids, and the functional assessment of variants associated with 28-day sepsis survival. [file 13054_2022_4208_MOESM1_ESM.pdf]

Supplementary material

A genome-wide association study of survival in patients with sepsis

Tamara Hernandez-Beeftink (MSc), Beatriz Guillen-Guio (PhD), Jose M. Lorenzo-Salazar (MSc), Almudena Corrales (LT), Eva Suarez-Pajes (MSc), Rui Feng (PhD), Luis A. Rubio-Rodríguez (MScEng), Megan L Paynton (PhD), Raquel Cruz (PhD), M. Isabel García-Laorden (PhD), Miryam Prieto-González (MD), Aurelio Rodríguez-Pérez (MD, PhD), Demetrio Carriedo (MD), Jesús Blanco (MD), Alfonso Ambrós (MD), Elena González-Higueras (MD), Elena Espinosa (MD, PhD), Arturo Muriel (MD, PhD), Eduardo Tamayo (MD), María M Martin (MD), Leonardo Lorente (MD), David Domínguez (MD, PhD), Abelardo García de Lorenzo (MD, PhD), Heather M. Giannini (MD, MSc), John P. Reilly (MD, MSc), Tiffanie K. Jones (MD, MSc), José M. Añón (MD, PhD), Marina Soro (MD), Ángel Carracedo (MD, PhD), Louise V. Wain (PhD), Nuala J Meyer (MD, MSc), Jesús Villar (MD, PhD), Carlos Flores (PhD) and the Genetics of Sepsis (GEN-SEP) Network.

Table of contents

Supplementary Methods **3**

GEN-SEP study population and samples 3

MESSI study population 3

Genotyping and quality control 3

Genome-wide association study of 28-day sepsis survival 4

Index event bias assessment 4

Annotation of the functional effects of associated variants and related genes 5

Gene expression of related genes 5

Association of polygenic risks of sepsis with the 28-day sepsis survival 6

Replication of genes from previous sepsis mortality GWAS 7

Supplementary References **7**

Supplementary Figures **10**

Supplementary Figure 1. Principal component analysis 10

Supplementary Figure 2. Quantile-quantile plot 11

Supplementary Figure 3. Regional plots for the genome-wide significant variants 12

Supplementary Figure 4. Kaplan-Meier 28-day survival plots 13

Supplementary Figure 5. Manhattan plot of sepsis 28-day survival association study results at the HLA region 14

Supplementary Figure 6. Heatmap plot of related genes across specific cell types 17

Supplementary Figure 7. Boxplot of *SAMD9* gene expression values GSE54514 and GSE65682 17

Supplementary Figure 8. Boxplot of *SAMD9* gene expression values in GSE32707 17

Supplementary Figure 9. Polygenic risk score (PRS) model fitting 18

Supplementary Tables **19**

Table S1. Relevant demographic and clinical features of cases and controls used for the GWAS of sepsis risk. 19

Table S2. Previous candidate genes associated with sepsis risk 20

Table S3. Demographic and clinical features of GEN-SEP patients 21

Table S4. Prioritized independent SNPs 22

Table S5. Results for the 28-day sepsis survival association of the significant SNPs after correcting for the index bias event. 23

Table S6. Association results with 28-day mortality and its percentage of variation explained by the SNPs dosages and PRS 23

Table S7. Association results of the sentinel variants from other sepsis mortality GWAS studies 25

Table S8. Nominally significant results for the classical HLA alleles and amino acids in GEN-SEP. 26

Table S9. Functional assessment of variants associated with 28-day sepsis survival 27

Supplementary Methods

GEN-SEP study population and samples

The GEN-SEP study [1], used for the first stage, is a national, multicenter, observational study conducted in a Spanish network of post-surgical units and ICUs recruiting unrelated adult (>18 years old) patients of European ancestry. This cohort included patients admitted in the following hospitals from Spain: Hospital Universitario de Canarias, Tenerife; Hospital Universitario NS de Candelaria, Tenerife; Hospital Universitario Río Hortega, Valladolid; Hospital Universitario Dr. Negrin, Gran Canaria; Hospital General de Ciudad Real, Ciudad Real; Complejo Hospitalario Universitario de León, León; Hospital Virgen de la Luz, Cuenca; Complejo Hospitalario Universitario de Santiago de Compostela, Santiago de Compostela; Fundació Althaia-Manresa, Barcelona; Hospital Clinic, Barcelona; Hospital Clínico de Valladolid; Hospital La Paz, Madrid; Hospital Fundación Jiménez Díaz, Madrid; Hospital del Bierzo, Ponferrada; Hospital General Río Carrión, Palencia, and Hospital Virgen de la Concha, Zamora. All participants or their representatives gave written informed consent, and the study was approved by the Ethics Committee for Drug Research from the Hospital Universitario de Canarias (Code: CHUNSC_2018-16).

Illustra™ blood genomicPrep Mini Spin Kit was used to purify DNA from peripheral blood for GEN-SEP samples and the concentration was measured on the Qubit 3.0 fluorometer with the dsDNA HS Assay kit (Thermo Fisher Scientific).

MESSI study population

Subjects from MESSI study were eligible if they were admitted to the medical ICU with infection-related organ dysfunction consistent with severe sepsis by Sepsis-2 criteria to maintain consistency with cohort initiation [2]. More than 95% of subjects met Sepsis-3 criteria also, but organ dysfunction beyond that captured by the sequential organ failure score was permitted. Subjects were excluded for chronic critical illness based on residence in a long-term acute care hospital, for prior enrollment to the cohort, or if they desired only palliative measures on ICU admission.

Genotyping and quality control

Genotyping in the GEN-SEP cohort was performed using the Axiom Genome-Wide Human CEU 1 array (Thermo Fisher Scientific) in the National Genotyping Center (CeGen), Universidad de Santiago de Compostela Node, Spain. The variant calling was achieved following the quality controls recommended by the manufacturer with AffyPipe v2.10.0 [3]. Subsequently, the genotyping quality controls were performed with PLINK v1.07 [4] and R v3.6.0 to filter out samples with genotype call rate (CR) <95% and with evidence of relatedness with other individuals (PIHAT>0.2) (**Figure 1**). Single nucleotide polymorphisms (SNPs) from mitochondrial DNA, chromosome Y, or with a genotyping CR <95%, a minor allele frequency (MAF) <0.01, or deviating from Hardy-Weinberg equilibrium (HWE) expectations ($p<1.0x10^{-6}$) were excluded from the study (**Figure 1**). PLINK v1.90 [5] was used to calculate the main axis of genetic

variation based on 115,611 (1st recruitment period) or 114,420 SNPs (2nd recruitment period) using principal component (PC) analyses (**Figure S1**).

Variant imputation in the GEN-SEP study was performed with the Michigan Imputation Server using the Haplotype Reference Consortium panel (v1.1 2016) [6]. To obtain a better coverage of the Human Leukocyte Antigen (HLA) region, we imputed the classic HLA alleles and amino acids using SHAPEIT v2.837 [7-8] for phasing haplotypes and Impute2 v2.3.2 [9] using the T1DGC HLA Reference Panel with SNP2HLA [10]. Variants with MAF <0.01 or with a low imputation quality (Rsq <0.3) were excluded from the analysis. The X chromosome was analysed separately for men and women, following all quality controls described above, although with SNP filtering based on HWE deviations calculated from females, and then meta-analysed.

In MESSI, SNPs were genotyped using the Axiom TxArray v.1 (Thermo Fisher Scientific). SNPs were filtered out if they were on sex chromosomes, had a MAF <0.01, deviated from HWE ($p<1.0x10^{-6}$), or had a genotyping call rate <95%. Pre-phasing and variant imputation were conducted on Michigan Imputation Server using Minimac3 v1.0.5 or Minimac4 v1.5.7 and The 1000 Genomes Project Phase 1 v3 CEU or CAAPA African American Panel as the reference panel.

Genome-wide association study of 28-day sepsis survival

In the first stage, we used the gwasurvivr v1.5.0 [11] package in R to test genetic associations. The meta-analysis of the two GEN-SEP recruitment periods was performed with METASOFT v2.0.1 [12]. The independence of prioritized variants was assessed by linkage disequilibrium (LD)-based clumping using PLINK v1.90 [5].

Finally, in the second stage, the meta-analysis from the GEN-SEP and MESSI studies was performed with METASOFT v2.0.1 [12].

To assess the variance in the outcome explained by the sentinel SNPs alone, we performed a logistic regression to test association with 28-day mortality adjusting by sex, age, and the two first principal components to obtain the Nagelkerke's statistic (R^2). We used the same approach but instead using a combined (PRS) score of the dosages from three leading SNPs to assess the variance in the outcome explained by the three SNPs together.

Index event bias assessment

Since we know that case-only analyses can be biased by variants that are associated with disease risk, we used the Index Event Bias correction [13]. This method uses effect sizes and standard errors for predictors of an index trait and a subsequent trait. This function adjusts the statistics for the subsequent trait for selection bias through the index trait. Thus, we used the results from the sepsis risk study to use the beta and standard error values and apply the method described above. Using a total of 112,363 independent SNPs and the SIMEX method (with 1,000 simulations performed in each stage of the Simex adjustment),

we obtained a maximal coefficient of 0.0116 that was used to correct the effect size and the *p*-value of the top hits from our survival GWAS.

Annotation of the functional effects of associated variants and related genes

LDproxy module from LDLink v4.1.0 was used to obtain proxies of the sentinel variants in European reference data [14]. Then, we used Genotype-Tissue Expression Project (GTEx) v7 datasets to obtain the local expression quantitative trait loci (eQTLs) [15]. HaploReg v4.1 [16], RegulomeDB v2.0.3 [17], and The Open Targets Post-GWAS portal [18] were used to annotate their regulatory potential and to rank their functional roles. To quantify the deleterious effects of noncoding variation, we used SNPDelScore [19]. We also analyzed long-distance genomic interactions with Capture Hi-C Plotter (CHiCP) [20]. Moreover, to assess the clinical interpretation of genetic variants we used InterVar, VarSome and ClinVar, as well, LoFtool score to evaluate the gene intolerance based on the ratio of LoF (stop-gain, splice site disrupting, frameshift) mutations to synonymous variants from the Exome Aggregation Consortium (ExAC) dataset. The Variant Effect Predictor (VEP) toolset from Ensembl was used to annotate and predict coding and non-coding regions and we used DeepSEA for a functional significance score prediction. In addition, PhenoScanner v2 was used to facilitate the cross-referencing of genetic variants with a broad range of phenotypes.

Finally, to evaluate the functional and gene targets for the non-coding RNA genes, we assessed the LncRNA2Target database [21], the LncRNA and Disease Database (version 2.0) [22], the miRTarBase Release 8.0 [23] and the Kyoto Encyclopedia of Genes and Genomes (KEGG).

Gene expression of related genes

To assess differential gene expression of the genes near the sentinel variants, we accessed the public gene expression data GSE54514 [24] and GSE65682 [25], available in the Gene Expression Omnibus (GEO) repository. GSE54514 contains transcriptomic information for healthy donors (n=18), and from sepsis survivors (n=26) and non-survivors (n=9) [24], whereas GSE65682 contains transcriptomic information for sepsis survivors (n=184) and non-survivors (n=48) [25]. Moreover, we accessed another expression microarray data set (GSE32707) [26] from sepsis patients (n=30) and patients with ARDS characterization (n=18) to assessed whether ARDS could be an alternative explanation of the gene expression observed differences. A two-sample t-test was used for comparisons, considering a False Discovery Rate (FDR) (q-value) to declare significance while adjusting for comparisons [27].

Likewise, we have also leveraged the data from 28 control subjects from the IPF Cell Atlas datasets (https://p2med.shinyapps.io/IPFCellAtlas) [28] to explore the transcriptional patterns of the three genes linked to the top SNPs in specific cell types and to improve functional inferences.

Association of polygenic risks of sepsis with the 28-day sepsis survival

Polygenic risk scores (PRS) allow for examining the cumulative effect of many genetic variants to be studied. For each individual, the PRS was calculated as the number of risk alleles carried, multiplied by the effect size of the variant, summed across all variants included in the score, i.e.:

$$\mathrm{PRS}_j = \sum_{i=1}^{n} \beta_i X_{ij}$$

where βi is the log(OR) of variant *i*, *Xij* is the genotype of variant *i* for person *j* and n is the number of variants included in the PRS. We investigated if the polygenic component of sepsis susceptibility was associated with 28-day sepsis survival using PRSice v1.25 software [29].

Given the lack of GWAS of sepsis risk in the literature, we firstly obtained genome-wide information of sepsis susceptibility comparing the genetic data from the 832 sepsis cases with available information from the GEN-SEP to 1,443 population controls. The controls declared having two generations of ancestors born in Spain and no personal or family history of infectious, cancerous, mental, cardiovascular or immunological diseases. The data from all these controls were used and described in previous studies [30-31].

For the controls, the array, quality controls, and imputation steps were equivalent to those of the GEN-SEP.

For this GWAS of sepsis susceptibility, association analyses were performed with logistic regression models using EPACTS v3.2.6 [32], adjusting for sex, age, and the two main PCs (not resulting in major systematic deviations from the null; λ=1.04).

For the PRS analyses, a total of 370,316 independent variants were selected after LD-clumping ($r^2 \leq 0.1$). We repeated this by excluding all variants within ±500 kb of the candidate genes that were associated with sepsis susceptibility in more than 20 publications (**Table S2**) according to the Public Health Genomics and Precision Health Knowledge Base (v6.2.3) database records. In this case, 368,724 independent variants were used. We also repeated this by excluding all variants within ±500 kb of the sentinel variants (rs4957796, rs117983287, rs143356980, and rs368584) that were associated with sepsis mortality in previous GWAS [33-36], leaving a total of 369,743 independent variants for further analysis.

Then, from the different PRS models, we included those variants that met a certain *p*-value threshold and varied this threshold to investigate the effect of including more variants in it. The PRS was tested to identify whether it was associated with 28-day sepsis survival, adjusting for sex, age, and two main PCs. We used Cox regressions with the 'survival' package in R v3.6.3 and the previously suggested threshold of $p<0.001$ [29] for defining a significant PRS (**Figure S7**).

Replication of genes from previous sepsis mortality GWAS

We evaluated whether the sentinel variants (rs4957796, rs117983287, rs143356980, and rs368584) previously associated with sepsis mortality in other GWAS [33-36] were associated with 28-day sepsis survival in the GEN-SEP cohort. Complementarily, we extracted ±50 kb of the regions around the genes where these variants reside (*CISH, FER*, *VPS13A*, and *COL4A2*) and the Genetic Type I error calculator (GEC) was used to estimate the number of independent SNPs for these four regions. This supported the existence of 1424 independent variants in Europeans considering the four regions together. Thus, we established the significance threshold at p=3.51x10^{-5} for this analysis.

Supplementary References

1. Guillen-Guio B, Lorenzo-Salazar JM, Ma SF, et al. (2020). Sepsis-associated acute respiratory distress syndrome in individuals of European ancestry: a genome-wide association study. Lancet Respir Med. 8(3):258-266.
2. Reilly JP, Wang F, Jones TK, et al. (2018). Plasma angiopoietin-2 as a potential causal marker in sepsis-associated ARDS development: evidence from Mendelian randomization and mediation analysis. Intensive Care Med. 44(11):1849-1858.
3. Nicolazzi EL, Iamartino D, Williams JL. (2014). AffyPipe: an open-source pipeline for Affymetrix Axiom genotyping workflow. Bioinformatics. 30(21):3118-9.
4. Purcell S, Neale B, Todd-Brown K, et al. (2007). PLINK: a tool set for whole-genome association and population-based linkage analyses. Am J Hum Genet. 81(3):559-75.
5. Chang CC, Chow CC, Tellier LC, Vattikuti S, Purcell SM, Lee JJ. (2015). Second-generation PLINK: rising to the challenge of larger and richer datasets. Gigascience. 4:7.
6. Loh PR, Danecek P, Palamara PF, et al. F. (2016). Reference-based phasing using the Haplotype Reference Consortium panel. Nat Genet. 48(11):1443-1448.
7. Delaneau O, Coulonges C, Zagury JF. (2008). Shape-IT: new rapid and accurate algorithm for haplotype inference. BMC Bioinformatics. 9:540.
8. Delaneau O, Marchini J; 1000 Genomes Project Consortium; 1000 Genomes Project Consortium. (2014). Integrating sequence and array data to create an improved 1000 Genomes Project haplotype reference panel. Nat Commun. 5:3934.
9. Howie BN, Donnelly P, Marchini J. (2009). A flexible and accurate genotype imputation method for the next generation of genome-wide association studies. PLoS Genet. 5(6):e1000529.
10. Jia X, Han B, Onengut-Gumuscu S, et al. (2013). Imputing amino acid polymorphisms in human leukocyte antigens. PLoS One. 8(6):e64683.
11. Rizvi AA, Karaesmen E, Morgan M, et al. P. (2019). gwasurvivr: an R package for genome-wide survival analysis. Bioinformatics. 35(11):1968-1970.
12. Han B, Eskin E. (2011). Random-effects model aimed at discovering associations in meta-analysis

of genome-wide association studies. Am J Hum Genet. 88(5):586-98.

13. Dudbridge F, Allen RJ, Sheehan NA, et al. (2019). Adjustment for index event bias in genome-wide association studies of subsequent events. Nat Commun. 10(1):1561.

14. Machiela MJ, Chanock SJ. (2015). LDlink: a web-based application for exploring population-specific haplotype structure and linking correlated alleles of possible functional variants. Bioinformatics. 31(21):3555-7.

15. GTEx Consortium. (2013). The Genotype-Tissue Expression (GTEx) project. Nat Genet. 45(6):580-5.

16. Ward LD, Kellis M. (2012). HaploReg: a resource for exploring chromatin states, conservation, and regulatory motif alterations within sets of genetically linked variants. Nucleic Acids Res. 40(Database issue):D930-4.

17. Boyle AP, Hong EL, Hariharan M, et al. (2012). Annotation of functional variation in personal genomes using RegulomeDB. Genome Res. 22(9):1790-7.

18. Peat G, Jones W, Nuhn M, et al. (2020). The open targets post-GWAS analysis pipeline. Bioinformatics. 36(9):2936-2937.

19. Alvarez RV, Li S, Landsman D, Ovcharenko I. (2018). SNPDelScore: combining multiple methods to score deleterious effects of noncoding mutations in the human genome. Bioinformatics. 34(2):289-291.

20. Schofield EC, Carver T, Achuthan P, et al. (2016). CHiCP: a web-based tool for the integrative and interactive visualization of promoter capture Hi-C datasets. Bioinformatics. 32(16):2511-3.

21. Jiang Q, Wang J, Wu X, et al. (2015). LncRNA2Target: a database for differentially expressed genes after lncRNA knockdown or overexpression. Nucleic Acids Res. 43(Database issue):D193-6.

22. Bao Z, Yang Z, Huang Z, et al. (2019). LncRNADisease 2.0: an updated database of long non-coding RNA-associated diseases. Nucleic Acids Res. 47(D1):D1034-D1037.

23. Huang HY, Lin YC, Li J, et al. (2020). miRTarBase 2020: updates to the experimentally validated microRNA-target interaction database. Nucleic Acids Res. 48(D1):D148-D154.

24. Parnell GP, Tang BM, Nalos M, et al. (2013). Identifying key regulatory genes in the whole blood of septic patients to monitor underlying immune dysfunctions. Shock. 40(3):166-74.

25. Scicluna BP, Klein Klouwenberg PM, van Vught LA, et al. (2015). A molecular biomarker to diagnose community-acquired pneumonia on intensive care unit admission. Am J Respir Crit Care Med. 192(7):826-35.

26. Dolinay T, Kim YS, Howrylak J, et al. (2012). Inflammasome-regulated cytokines are critical mediators of acute lung injury. Am J Respir Crit Care Med. 185(11):1225-34.

27. Benjamini Y, Hochberg, Y. (1995). Controlling the false discovery rate: a practical and powerful approach to multiple testing. Journal of the Royal Statistical Society: Series B (Methodological)

57:289–300.

28. Neumark N, Cosme C Jr, Rose KA, Kaminski N. (2020) The Idiopathic Pulmonary Fibrosis Cell Atlas. Am J Physiol Lung Cell Mol Physiol. 319(6):L887-L893.

29. Euesden J, Lewis CM, O'Reilly PF. (2015). PRSice: Polygenic Risk Score software. Bioinformatics. 31(9):1466-8.

30. Barreto-Luis A, Pino-Yanes M, Corrales A, et al. (2016). Genome-wide association study in Spanish identifies ADAM metallopeptidase with thrombospondin type 1 motif, 9 (ADAMTS9), as a novel asthma susceptibility gene. J Allergy Clin Immunol. 137(3):964-6.

31. Guillen-Guio B, Hernández-Beeftink T, Marcelino-Rodríguez I, et al. (2020). Admixture mapping of asthma in southwestern Europeans with North African ancestry influences. Am J Physiol Lung Cell Mol Physiol. 318(5):L965-L975.

32. Kang HM. (2016). EPACTS (Efficient and Parallelizable Association Container Toolbox), http://genome.sph.umich.edu/wiki/EPACTS.

33. Rautanen A, Mills TC, Gordon AC, et al. (2015). Genome-wide association study of survival from sepsis due to pneumonia: an observational cohort study. Lancet Respir Med. 3(1):53-60.

34. Scherag A, Schöneweck F, Kesselmeier M, et al. (2016). Genetic Factors of the Disease Course after Sepsis: A Genome-Wide Study for 28Day Mortality. EBioMedicine. 12:239-246.

35. Rosier F, Brisebarre A, Dupuis C, et al. (2021). Genetic Predisposition to the Mortality in Septic Shock Patients: From GWAS to the Identification of a Regulatory Variant Modulating the Activity of a CISH Enhancer. Int J Mol Sci. 22(11):5852.

36. D'Urso S, Rajbhandari D, Peach E, et al. Septic Shock: A Genomewide Association Study and Polygenic Risk Score Analysis. Twin Res Hum Genet. 2020; 23(4):204-213.

37. Choi W, Luo Y, Raychaudhuri S, Han B. (2021). HATK: HLA analysis toolkit. Bioinformatics. 37(3):416-418.

Supplementary Figures

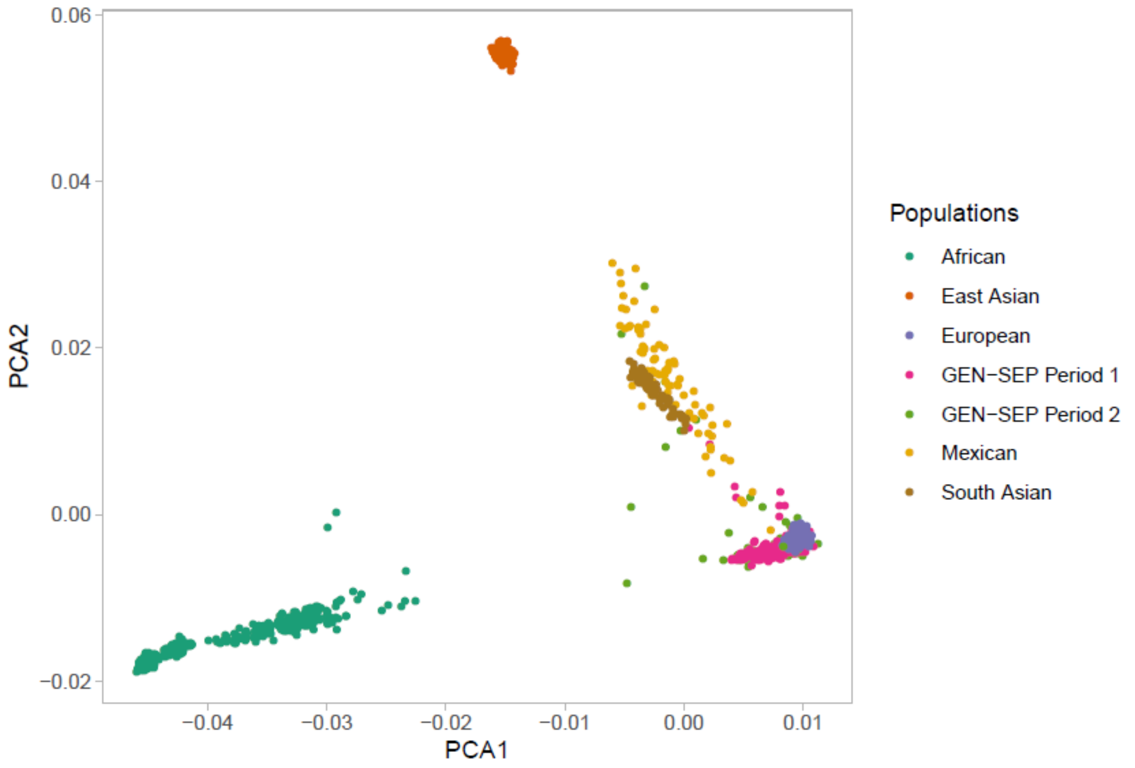

Supplementary Figure 1*. Principal component analysis. Plot of the first two principal components (PCs) of genetic variation of GEN-SEP individuals analysed in the first stage projected on the HapMap3 reference dataset.*

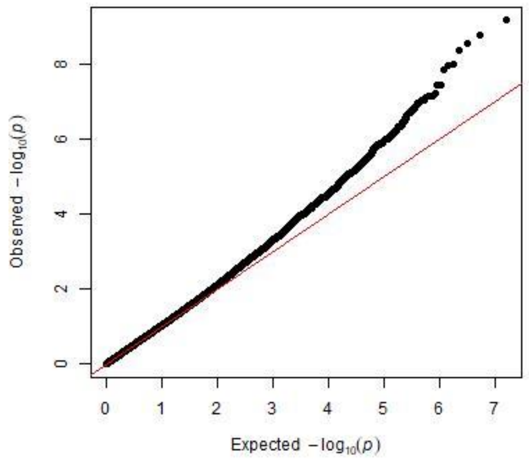

Supplementary Figure 2. *Quantile-quantile plot of the observed versus expected -log10 p-values of the association results of first stage.*

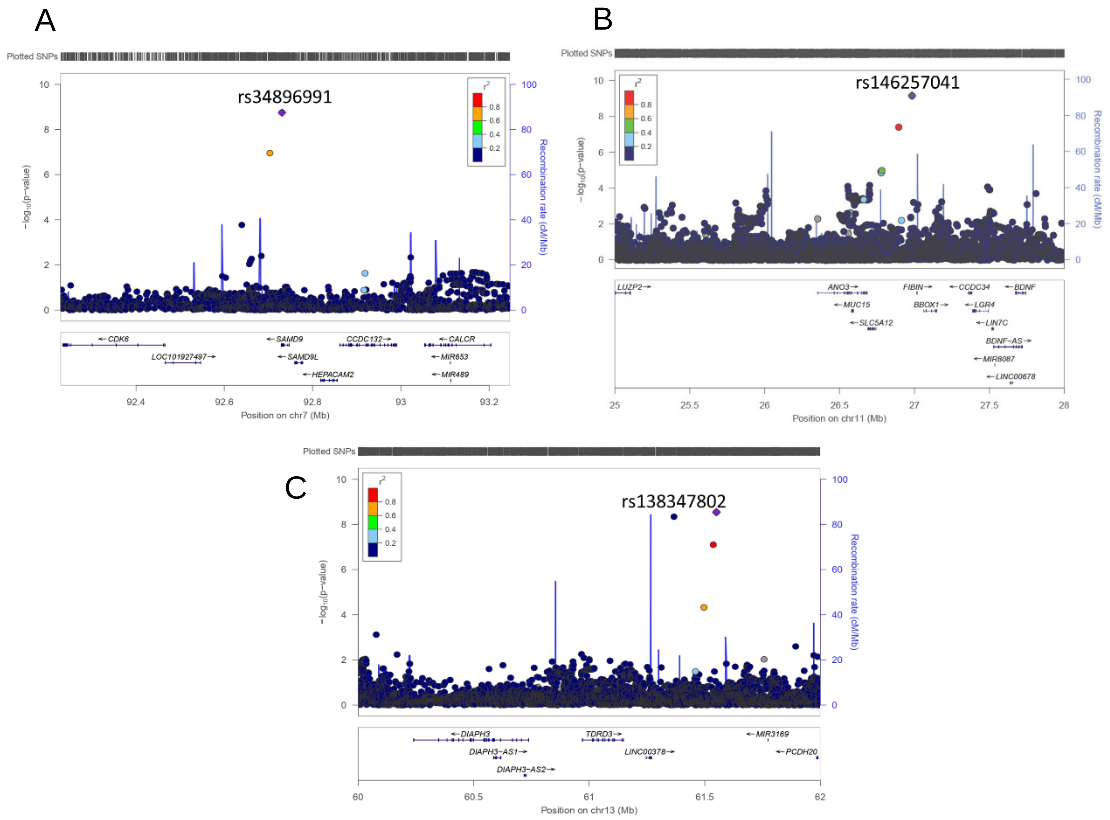

***Supplementary Figure 3**. Regional plots for the genome-wide significant variants rs34896991 (A), rs146257041 (B), and rs138347802 (C) in the first stage. The x-axis shows the hg19 genomic positions and the left y-axis represents the -log10(p-value). Estimated recombination rates (light blue line) are also plotted on the right y-axis. Linkage disequilibrium values with respect to the sentinel variant (indicated) based on pairwise r2 values in Europeans from the 1000 Genomes Project are shown. Obtained with LocusZoom.*

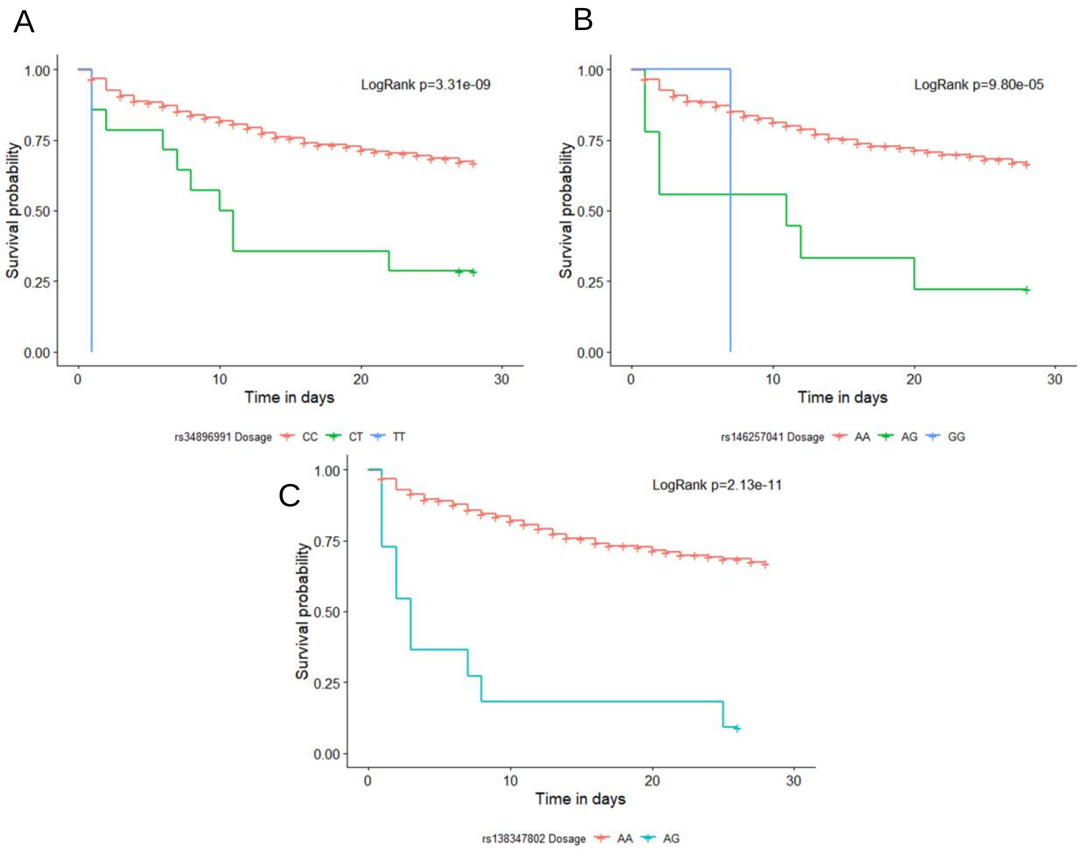

Supplementary Figure 4*. Kaplan-Meier 28-day survival plots and the log-rank p-value for the genotypes of the genome-wide significant variants on the first stage: A) rs34896991 (in SAMD9); B) rs146257041 (in SLC5A12\FIBIN); and C) rs138347802 (in LINC00378\MIR3169).*

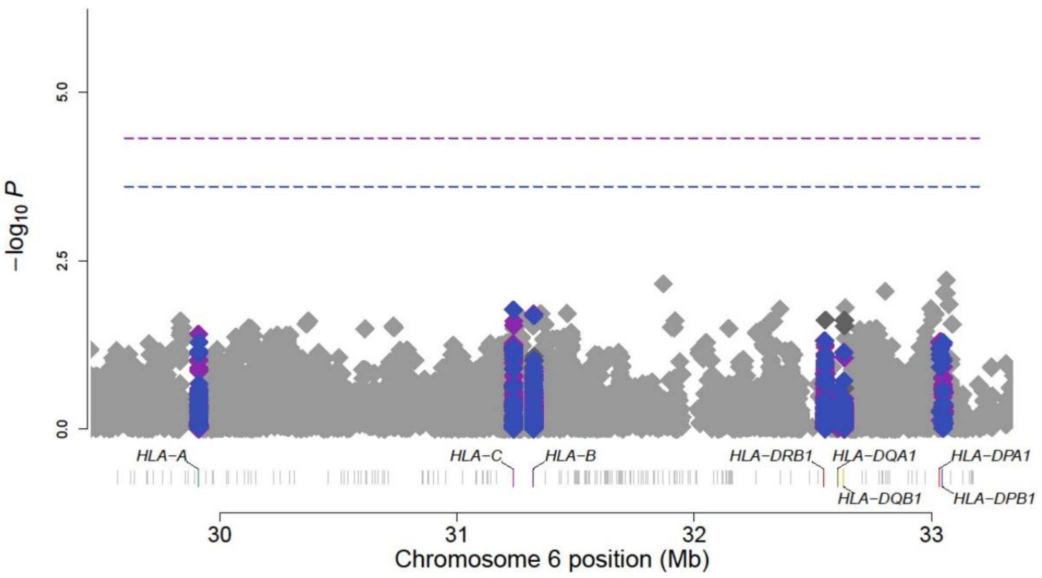

Supplementary Figure 5. *Manhattan plot of sepsis 28-day survival association study results at the HLA region. Highlighted in blue are the classical HLA alleles results, in purple the amino acids results, and in grey the GWAS results of the SNPs (chr6: 28,477,797-33,448,354). Horizontal lines indicate the significance threshold for the different tests: In blue, the threshold for classical HLA alleles $p=2.49x10^{-4}$; and in purple, the threshold for amino acids $p=4.83x10^{-5}$. The x-axis shows genomic position and y-axis represents the significance (-log10[p-value]). The association results were represented using the HLA Analysis Tool-Kit (HATK) [37].*

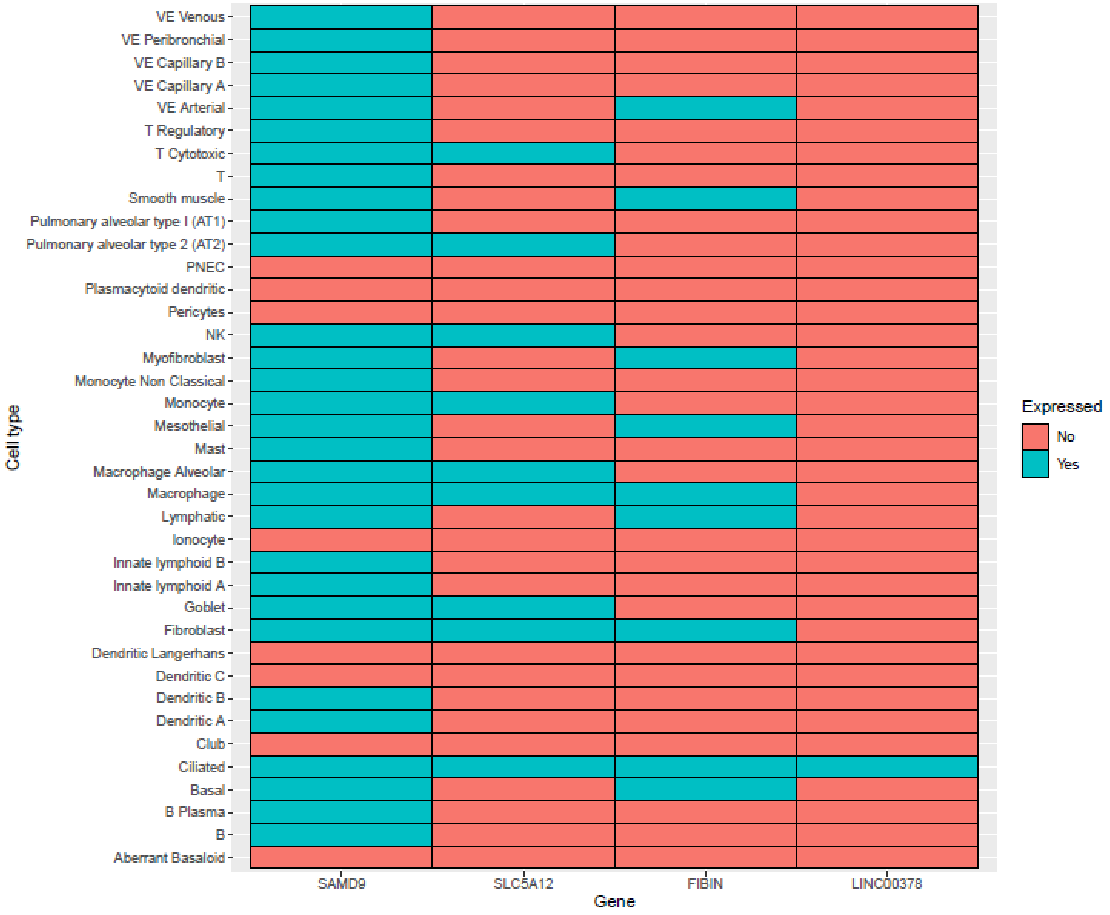

Supplementary Figure 6*. Transcriptional expression of the genes near the sentinel variants across specific cell types from control subjects in the IPF Cell Atlas.*

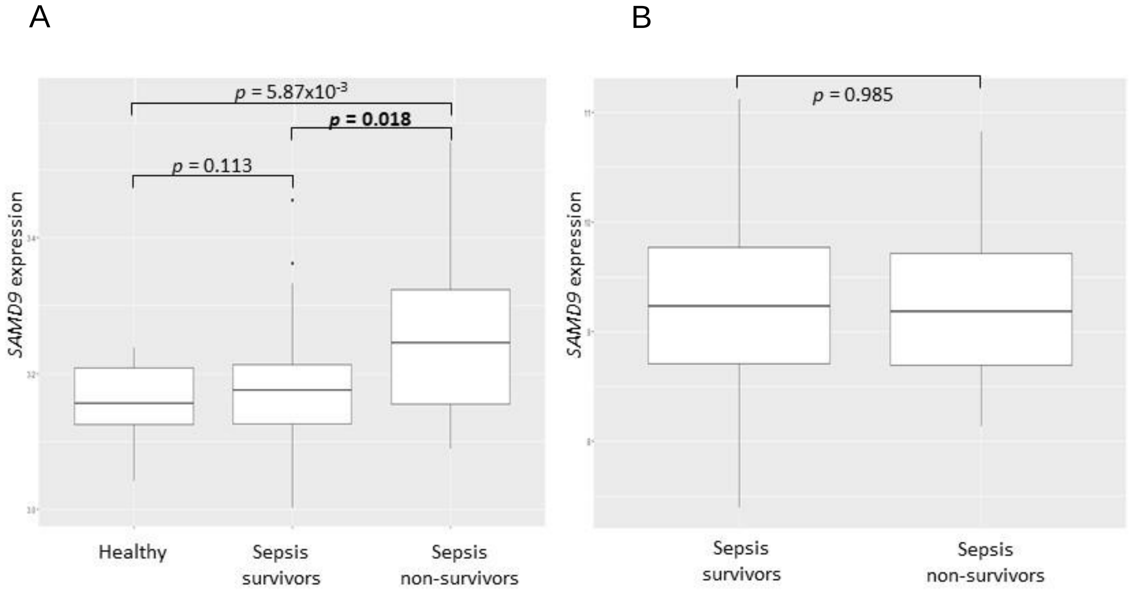

Supplementary Figure 7. *Boxplot of SAMD9 gene expression values in A) GSE54514 and B) GSE65682. Significance was calculated by two-sample t-test.*

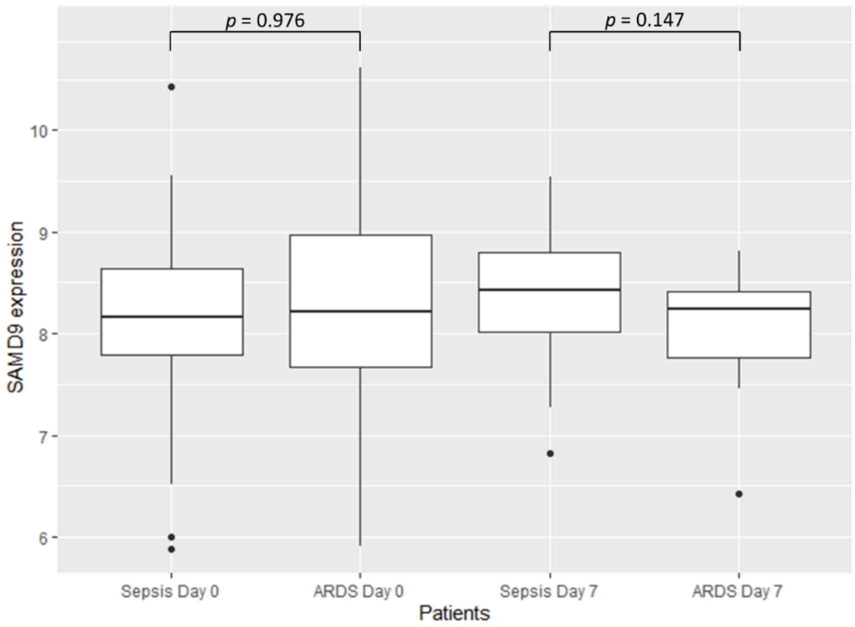

Supplementary Figure 8. *Boxplot of SAMD9 gene expression values in GSE32707. Significance was calculated by two-sample t-test.*

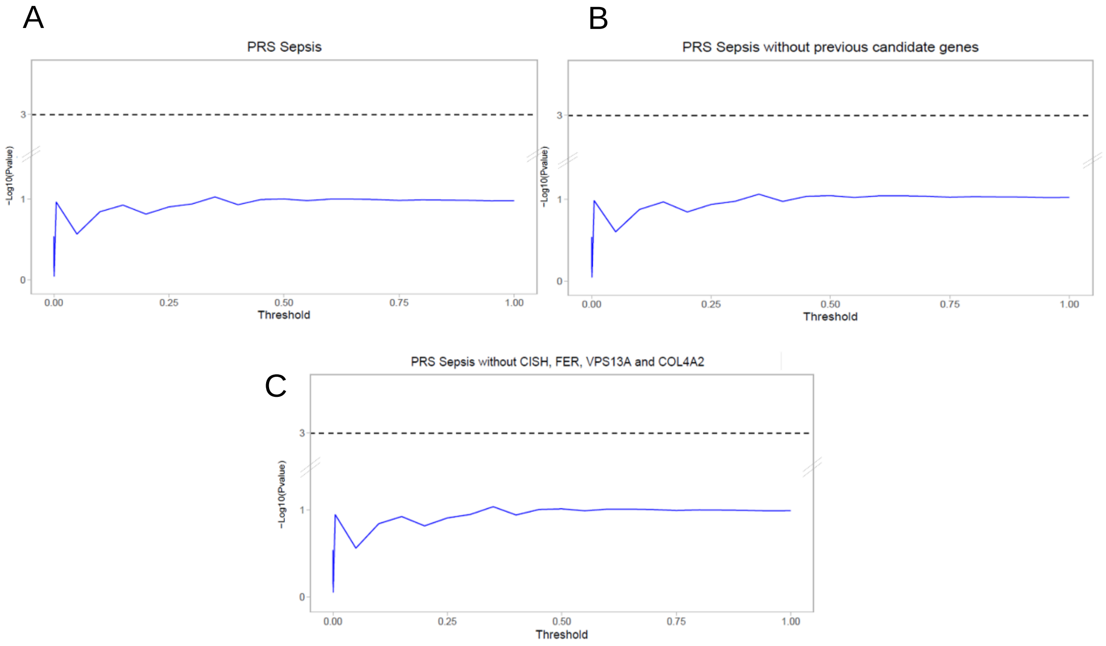

Supplementary Figure 9. *Polygenic risk score (PRS) model fitting and strength of association in the GEN-SEP patients with available mortality data at 28 days after sepsis diagnosis using the Cox regression p-values. The x-axis shows the threshold p-value used for selecting the variants to be included in the risk score model. The y-axis shows the significance (−log10 (p-value)) for the association of the risk score models with 28-day survival for each threshold used. Horizontal lines indicate the significance threshold. A) All-inclusive PRS model fitting. B) PRS models fitting excluding variants (+/- 500 kb) linked to sepsis risk based on candidate gene studies (see* ***Table S2****). C) PRS model fitting excluding the variants (and +/- 500 kb proxies) of sepsis mortality genes (FER, VPS13A, CISH, and COL4A2) described in previous GWAS.*

Supplementary Tables

Table S1. **Relevant demographic and clinical features of cases and controls used for the GWAS of sepsis risk after quality controls.**

	Cases (N = 832)	Controls (N = 1,443)	*p*-value*
Sex, % male (N)	63 (524)	49 (703)	< 0.0001
Age, mean years ± SD	63 ± 15	47 ± 14	< 0.0001
BMI, mean kg/m2 ± SD	28 ± 6	NA	NA
SAPS, mean ± SD	47 ± 15	NA	NA
APACHE II (8h), mean ± SD	20 ± 7	NA	NA
SOFA, mean ± SD	8 ± 4	NA	NA
Comorbidities[$], % (N)	46 (326)	NA	NA
ARDS, % (N)	38 (318)	NA	NA
Hospital mortality, % (N)	35 (287)	NA	NA
ICU mortality, % (N)	28 (228)	NA	NA
Days in Hospital, mean ± SD	38 ± 50	NA	NA
Days in ICU, mean ± SD	17 ± 24	NA	NA
Sepsis of pulmonary origin, % (N)	35 (264)	NA	NA
Pathogen, % (N)			
Gram-positive	26 (150)	NA	NA
Gram-negative	37 (209)	NA	NA
Others[+]	28 (158)	NA	NA

*Sex comparison was assessed by a chi-square test. Age was compared using the Mann–Whitney U-test.
[$]Comorbidities include cancer, age >80 years, hepatopathy, valvular disease, immunodeficiency, severe brain damage, morbid obesity, chronic disease, autoimmune disease, pregnancy, myopathy, pneumonia, and serious recurrent infections.
[+]Others include both (Gram-positive and Gram-negative), fungi, virus and polymicrobial.
APACHE II, Acute Physiology and Chronic Health Evaluation II; ARDS, Acute Respiratory Distress Syndrome; BMI, Body Mass Index; ICU, Intensive Care Unit; SAPS, Simplified Acute Physiology Score II; SOFA, Sequential Organ Failure Assessment.

**Table S2. Previous candidate genes associated with sepsis risk obtained from the Public Health Genomics and Precision Health Knowledge Base (v6.2.3) database.**				
**Associated Gene**	**Total Publications**	**Gene name**	**Genomic locations (GRCh37/hg19)**	**Position +/- 500 kb**
TNF	102	Tumour Necrosis Factor	chr6:31,543,344-31,546,113	chr6:31,043,344-32046113
IL6	65	Interleukin 6	chr7:22,765,503-22,771,621	chr7:22,265,503-23,271,621
IL10	60	Interleukin 10	chr1:206,940,947-206,945,839	chr1:206,440,947-210,445,839
TLR4	53	Toll Like Receptor 4	chr9:120,466,460-120,479,768	chr9:119,966,460-120,979,768
CD14	42	Monocyte Differentiation Antigen CD14	chr5:140,011,313-140,013,286	chr5:139,511,313-140,513,286
MBL2	41	Mannose Binding Lectin 2	chr10:54,525,140-54,531,460	chr10:54,025,140-55,031,460
IL1B	40	Interleukin 1 Beta	chr2:113,587,328-113,594,480	chr2:113,087,328-114,094,480
TLR2	34	Toll Like Receptor 2	chr4:154,605,441-154,627,243	chr4:154,105,441-155,127,243
LTA	33	Lymphotoxin Alpha	chr6:31,539,831-31,542,101	chr6:31,039,831-32,042,101
SERPINE1	20	Serpin Peptidase Inhibitor, Clade E (Nexin, Plasminogen Activator Inhibitor Type 1)	chr7:100,770,370-100,782,547	chr7:100,270,370-101,282,547
IL1RN	22	Interleukin 1 Receptor Antagonist	chr2:113,864,791-113,891,593	chr2:113,364,791-114,391,593

**Table S3. Demographic and clinical features of GEN-SEP patients of the first stage GWAS of 28-day survival.**						
	1st GEN-SEP period			2nd GEN-SEP period		
	**Survivors (N=329)**	**Deaths (N=146)**	***p*-value***	**Survivors (N=177)**	**Deaths (N=35)**	***p*-value***
Sex, % male (N)	64 (212)	68 (100)	0.451	56 (100)	51 (18)	0.715
Age, mean years ± SD	62 ± 15	66 ± 13	4.93×10^{-3}	64 ± 15	73 ± 13	2.36×10^{-3}
BMI, mean ± SD	28 ± 6	26 ± 5	0.089	27 ± 6	26 ± 5	0.509
LISS, Mean ± SD	3 ± 2	4 ± 3	0.263	1 ± 2	1 ± 1	0.148
SAPS, mean ± SD	45 ± 13	53 ± 16	4.56×10^{-5}	50 ± 16	48 ± 26	0.892
APACHE II, mean ± SD	20 ± 7	25 ± 7	1.72×10^{-10}	17 ± 7	22 ± 7	2.64×10^{-4}
SOFA, mean ± SD	8 ± 4	9 ± 5	3.71×10^{-3}	7 ± 3	10 ± 4	7.21×10^{-4}
ARDS, % (N)	44 (145)	60 (87)	2.51×10^{-3}	13 (23)	26 (9)	0.096
Comorbidities[$], % (N)	41 (118)	52 (56)	0.063	39 (66)	46 (16)	0.606
Sepsis of pulmonary origin, % (N)	39 (125)	39 (57)	0.962	23 (40)	31 (11)	0.368
Days in Hospital, mean ± SD	45 ± 40	17 ± 12	2.23×10^{-17}	36 ± 49	16 ± 13	7.20×10^{-5}
Days in ICU, mean ± SD	22 ± 23	10 ± 7	1.22×10^{-7}	13 ± 30	9 ± 7	0.742
Pathogen, % (N)						
Gram-positive	30 (59)	32 (26)	0.794	21 (36)	15 (5)	0.561
Gram-negative	41 (81)	35 (28)	0.412	30 (52)	35 (12)	0.688
Others+	30 (59)	33 (27)	0.341	28 (49)	38 (13)	0.343

*Comparisons for sex, ARDS, comorbidities, sepsis of pulmonary origin, and pathogen were conducted by chi-square test. The rest of variables were compared using a Mann–Whitney U test.

[$]Includes: cancer, age >80 years, hepatopathy, valvular disease, immunodeficiency, severe brain damage, morbid obesity, chronic disease, autoimmune disease, pregnancy, myopathy, pneumonia, and serious recurrent infections.

[+]Includes: mixed Gram-positive and Gram-negative infection, fungi, virus, and polymicrobial.

APACHE II, Acute Physiology and Chronic Health Evaluation II; ARDS, acute respiratory distress syndrome; BMI, Body Mass Index; ICU, Intensive Care Unit; SAPS, Simplified Acute Physiology Score II; SOFA, Sequential Organ Failure Assessment.

Table S4. Prioritized independent SNPs from the first stage of the GWAS of 28-day sepsis survival.

						GEN-SEP (N=687)		MESSI (N=1,362) (European Americans)		MESSI (N=701) (African Americans)		Meta-analysis (N=2,750)	
**rsID**	**Position (Hg19)**	**A1/A2**	**Function**	**Gene**	**EAF**	**HR (95% CI)**	***p*-value**	**HR (95% CI)**	***p*-value**	**HR (95% CI)**	***p*-value**	**HR (95% CI)**	***p*-value**
rs114581095	3:36047297	C/T	Intergenic	*ARPP21\STAC*	0.016	6.07 (3.15-11.71)	$7.59x10^{-8}$	1.14 (0.91-1.43)	0.380	0.79 (0.39-1.58)	0.712	1.79 (1.38-6.35)	$7.84x10^{-6}$
rs114658749	3:195775144	C/T	Intergenic	*SDHAP1\TFRC*	0.062	3.05 (2.03-4.60)	$9.16x10^{-8}$	0.83 (0.55-1.26)	0.255	1.17 (0.51-2.64)	0.501	1.44 (1.22-3.02)	$5.56x10^{-6}$
rs76805442	5:84399852	G/A	Intergenic	*EDIL3\NBPF22P*	0.066	2.41 (1.71-3.39)	$4.37 x10^{-7}$	1.05 (0.83-1.32)	0.694	1.88 (0.99-3.58)	0.054	1.65 (1.33-3.05)	$3.39x10^{-6}$
rs73285901	7:25189764	G/A	Intronic	*C7orf31*	0.032	3.19 (2.03-5.01)	$4.76x10^{-7}$	0.99 (0.74-1.34)	0.968	0.89 (0.74-1.07)	0.199	1.37 (1.37-2.55)	$8.77x10^{-5}$
rs34896991	7:92730745	C/T	Exonic	*SAMD9*	0.018	4.75 (2.86-7.89)	$1.77x10^{-9}$	0.61 (0.29-1.28)	0.188	1.44 (0.46-4.50)	0.534	1.64 (1.37-6.78)	$4.92x10^{-8}$
rs146257041	11:26983813	A/G	Intergenic	*SLC5A12\FIBIN*	0.015	5.14 (3.06-8.65)	$7.04x10^{-10}$	NA	NA	0.89 (0.12-6.39)	0.911	4.59 (2.77-7.59)	$3.00x10^{-9}$*
rs138347802	13:61367197	A/G	Intergenic	*LINC00378\MIR3169*	0.014	8.33 (4.1-16.91)	$4.56x10^{-9}$	0.68 (0.3-1.52)	0.345	2.98 (0.95-9.38)	0.062	2.57 (1.82-13.03)	$4.44x10^{-8}$
rs113925942	13:93552176	T/C	Intergenic	*GPC5\LINC00363*	0.022	4.10 (2.44-6.88)	$9.63x10^{-8}$	1.10 (0.49-2.46)	0.813	NA	NA	2.21 (1.59-3.07)	$1.34x10^{-6}$*
rs183364907	14:58109460	A/T	Intronic	*SLC35F4*	0.011	7.40 (3.55-15.45)	$9.79x10^{-8}$	0.54 (0.22-1.3)	0.171	$2.88x10^{-7}$ (0-Inf)	0.992	2.02 (1.51-2.72)	$1.41x10^{-6}$
rs146077854	17:14555200	G/A	Intergenic	*HS3ST3B1\LOC101928475*	0.010	7.25 (3.36-15.67)	$4.65x10^{-7}$	NA	NA	NA	NA	NA	NA
rs1573332	21:39902441	T/A	Intronic	*ERG*	0.345	1.89 (1.48-2.40)	$2.41x10^{-7}$	0.96 (0.85-1.08)	0.485	NA	NA	1.33 (1.17-1.53)	$1.14x10^{-5}$*

*Meta-analysis performed between two studies.

A1: Ref allele; A2: Effect allele; EAF: Effect allele frequency; HR: Hazard ratio; CI: Confidence Interval; Position: chromosome and base pair.

Table S5. Results for the 28-day sepsis survival association of the significant SNPs after correcting for the index bias event.

			28-day survival model			Sepsis risk model			28-day survival with index event bias adjustment			
**Gene**	**rsID**	**A1/A2**	**Beta**	**SE**	***p*-value**	**Beta**	**SE**	***p*-value**	**Beta**	**SE**	**HR (95% CI)**	***p*-value**
SAMD9	rs34896991	C/T	1.558	0.259	1.77×10^{-9}	0.389	0.320	0.223	1.554	0.259	4.73 (2.85-7.86)	9.99×10^{-10}
SLC5A12\FIBIN	rs146257041	A/G	1.638	0.266	7.04×10^{-10}	0.675	0.355	0.057	1.630	0.266	5.10 (3.03-8.59)	4.39×10^{-10}
LINC00378\MIR3169	rs138347802	A/G	2.120	0.362	4.56×10^{-9}	0.202	0.405	0.617	2.117	0.362	8.31 (4.09-16.88)	2.40×10^{-9}

A1: Ref allele; A2: Effect allele; EAF: Effect allele frequency; HR: Hazard ratio.

Table S6. Association results with 28-day mortality and the percentage of variation explained by the three SNPs by separate or when combined in a PRS.

	OR (95% CI)	p-value	Nagelkerke (R^2)
rs34896991	1.50 (1.27-1.78)	$2.53x10^{-6}$	0.062
rs146257041	1.66 (1.35-2.03)	$1.25x10^{-6}$	0.064
rs138347802	1.72 (1.35-2.20)	$1.46x10^{-5}$	0.057
PRS	3.60 (2.47-5.24)	$4.63x10^{-11}$	0.090

Table S7. Association results of the sentinel variants from other sepsis mortality GWAS studies in GEN-SEP and the variant with the lowest *p*-value in the gene region in this study.

rsID	Study	Position (hg19)	A1/A2	Function	Gene	EAF	HR (95% CI)	*p*-value
rs143356980	Rosier et al.	3:50638812	C/T	Intergenic	*HEMK1\CISH*	0.013	1.42 (0.61-3.33)	0.419
rs78292306	GEN-SEP	3:50646863	G/A	Intronic	*CISH*	0.021	0.57 (0.23-1.41)	0.224
rs4957796	Rautanen et al.	5:108402140	T/C	Intronic	*FER*	0.161	1.09 (0.83-1.43)	0.550
rs115761658	GEN-SEP	5:108397843	G/A	Intronic		0.020	3.58 (1.83-7.03)	$2.05x10^{-4}$
rs117983287	Scherag et al.	9:80020874	C/A	Exonic	*VPS13A*	0.019	0.36 (0.09-1.48)	0.158
rs4745630	GEN-SEP	9:80023542	G/A	Intronic		0.286	1.39 (1.12-1.73)	$2.99x10^{-3}$
rs368584	D'Urso et al.	13:111145073	G/C	Intronic	*COL4A2*	0.422	1.02 (0.83-1.26)	0.817
rs447049	GEN-SEP	13:111143986	A/G	Intronic		0.242	2.00 (0.83-4.80)	$3.27x10^{-4}$

A1: Ref allele; A2: Effect allele; EAF: Effect allele frequency from Stage 1; HR: Hazard ratio.

**Table S8. Nominally significant (p<0.05) results for the classical HLA alleles and amino acids in GEN-SEP.**						
	**Gene**	**Allele/Variant***	**Genomic position (hg19)**	**Freq.**	***p*-value**	**HR (95% CI)**
Classical HLA alleles	*HLA-C*	03:03	6:31238192	0.965	0.017	0.57 (0.36-0.90)
	HLA-B	15:01	6:31323293	0.970	0.021	0.55 (0.33-0.91)
	HLA-DRB1	10	6:32552064	0.982	0.049	0.54 (0.29-1.00)
	HLA-DRB1	10:01	6:32552064	0.982	0.049	0.54 (0.29-1.00)
Amino acids	*HLA-C*	Arg91Gly	6:31347104	0.965	0.017	0.57 (0.36-0.90)
	HLA-B	Trp156	6:31432003	0.968	0.019	0.55 (0.33-0.91)
	HLA-B	--156	6:31432003	0.968	0.019	0.55 (0.33-0.91)
	HLA-C	Leu/Trp156	6:31346909	0.419	0.025	0.79 (0.64-0.97)
	HLA-C	Arg/Gln156	6:31346909	0.590	0.029	1.27 (1.02-1.57)
	HLA-A	Arg/Trp156	6:30019219	0.756	0.039	0.77 (0.61-0.99)
	HLA-DRB1	Pro231	6:32656010	0.982	0.049	0.54 (0.29-1.00)
	HLA-DRB1	Gln166	6:32657380	0.982	0.049	0.54 (0.29-1.00)
	HLA-DRB1	Arg30	6:32660058	0.982	0.049	0.54 (0.29-1.00)
	HLA-DRB1	Val31	6:32660055	0.982	0.049	0.54 (0.29-1.00)
	HLA-DRB1	Ala38	6:32660034	0.982	0.049	0.54 (0.29-1.00)
	HLA-DRB1	Tyr40Phe	6:32660028	0.982	0.049	0.54 (0.29-1.00)
	HLA-DRB1	Glu10	6:32660118	0.982	0.049	0.54 (0.29-1.00)

*Amino acid and position; Freq: Frequency of classical HLA allele or amino acids; HR: Hazard ratio.

Table S9. Functional assessment of variants associated with 28-day sepsis survival after meta-analysis.

	**rs34896991** (*SAMD9)*	**rs146257041** *(SLC5A12\FIBIN)*	**rs138347802** (LINC00378\MIR3169)
**Chromosome location**	chr7:92730745 (p.Ala1556Thr)	chr11:26983813 (intergenic)	chr13:61367197 (intergenic)
**Functional significance score** [DeepSEA $p \leq 0.05$]	5.87×10^{-2}	0.235	0.138
**Clinical interpretation of genetic variants**	Likely benign (InterVar); Benign (Varsome and ClinVar)	NA	NA
**LoFtool score < 0.1**	0.991	None	None
**CADD (scaled)**	10.8	1.61	6.73
**Variant Effect Predictor (**[VEP]**)**	1 feature: ENSG00000205413 (missense variant)	None	None
**RegulomedB rank (Score)**	Motif hit (6, 0.437)	Other (7, 0.184)	Other (7, 0.184)
**Enhancer histone marks** [HaploReg]	None	H3K4me1 (Mesench and Epithelial) and H3K27ac (Blood & Primary T helper memory cells from peripheral blood; Epithelial, Foreskin Keratinocyte Primary Cells skin and Muscle Colon Smooth Muscle).	None
**Promoter histone marks** [HaploReg]	H3K4me3 (Blood and T-cell; Mesench; Brain; Adipose; Heart; Muscle; Digestive; Fetal Lung and Liver)	None	H3K9ac (Breast Myoepithelial Primary Cells)
**DNAse** [HaploReg]	None	None	None
**Altered regulatory motifs** [HaploReg]	Egr-1,Pax-4	ERalpha-a	5 altered motifs (AP-1_disc6, DMRT2, Hoxa7_1, Nanog_disc4 and Pou2f2)
**Proteins bound** [HaploReg]	None	None	None
**CHICP**	None	NA	NA
**Open Targets Genetics** Top ranked genes based on the overall V2G score	0.27 (*SAMD9*)	0.14 (*SLC5A12*)	0.07 (LINC01442)
**dsQTL** [GTEX] Tissue-specific $p \leq 0.05$	None	None	None
**eQTL** [GTEX] Tissue-specific $p \leq 0.05$	Not significant	4 significant eQTLs were found for this variant in brain and testis	Not significant
**Score CAPE dsQTL >0.5**	None	None	None
**Score CAPE eQTL >0.5**	None	None	None
**PhenoScanner** $p \leq 0.05$	Allele T has been related with the cause of death of other specified degenerative diseases of the nervous system (p=8.62×10^{-11}).	NA	NA

CADD, Combined annotation dependent depletion; CAPE, Cellular dependent deactivating; CHiCP, capture HiC plotter; dsQTL, DNase I sensitivity quantitative trait loci; EGR-1, Early growth response protein 1; eQTL, expression quantitative trait loci; PAX-4, Paired box 4; and V2G, Assigning variants to genes.
